# Gas Poisoning in Mining and Other Industries

**Published:** 1914-12

**Authors:** 


**Gas Poisoning in Mining and other Industries.** By John Glaister, M.D., D.P.H., F.R.C.S.E., etc., and David Dale Logan, M.D., D.P.H. Pp. xi. 471. Edinburgh: E. & S. Livingstone. 1914. 10s. 6d. net.

At last we have a reasonably complete account of the voluminous literature in recent years upon the subject of carbon monoxide poisoning, a subject which has become of great importance owing to the immense increase in the use of "producer gas," and gas engines in place of steam, and to other industrial changes. The traveller by night through the Black Country no longer sees pillars of fire from the blast furnaces, for the gas is now covered in and carried off to drive the engines and even to work the mines. Unfortunately the furnace gas which contains perhaps 30 per cent. of CO, has a disastrous tendency to leak through tubes, earth, and even brickwork, with often fatal results. In mines the fire-damp, $CH_4$, and the black or choke-damp are in themselves chiefly harmful through the absence of oxygen; but explosions, fires, and blasting produce after-damp and white or sweet-damp, containing large quantities of the deadly carbon monoxide. It is curious that there should have been so much discussion as to whether this gas is poisonous in itself or merely from its excluding oxygen, for there can be little doubt of its intrinsic harmfulness when we consider its peculiar effect on respiration, so different from that of $CO_2$ and similar bodies, and the hemorrhages it causes in the brain and cord. The author describes at length the remarkable group of symptoms to which it gives rise, especially the sudden loss of muscular power, the heart failure, the neuritis, the bulbar and other nervous affections. Beside the central paralyses there are frequently peripheral ones, which are rarely symmetrical and most often right-sided. Trophic and vasomotor changes may occur without loss of motor power and sensation, or *vice versa*. As to treatment, we are warned of the necessity of forbidding exertion and maintaining body warmth, of keeping up artificial respiration for long periods, and of administering oxygen by a mask under some pressure. In a few cases venesection may be useful, as well as saline injections and transfusions.

A very full abstract is given of Mott's invaluable paper on "The Pathology of CO Poisoning," and a bibliography extending over eleven pages.

The arrangement of the material is perhaps hardly as clear as might be wished, but the wealth of detail collected from English and foreign sources is so great that one cannot be surprised. The book is of great value not only on its scientific side, but also for sanitary work and in connection with compensation cases.

